# Hemolytic Disease of the Fetus and Newborn in an Integrated Health Care System

**DOI:** 10.1055/a-2558-7891

**Published:** 2025-04-17

**Authors:** Michael J. Fassett, Nehaa Khadka, Fagen Xie, Jiaxiao Shi, Vicki Y. Chiu, Theresa M. Im, Sunhea Kim, Nana A. Mensah, Daniella Park, Carol Mao, Matthew Molaei, Iris Lin, Darios Getahun

**Affiliations:** 1 Department of Obstetrics and Gynecology, Kaiser Permanente West Los Angeles Medical Center, Los Angeles, California; 2 Department of Clinical Science, Kaiser Permanente Bernard J. Tyson School of Medicine, Pasadena, California; 3 Department of Research and Evaluation, Kaiser Permanente Southern California, Pasadena, California; 4 Janssen Global Services, LLC, Horsham, Pennsylvania; 5 Janssen Scientific Affairs, LLC, Horsham, Pennsylvania; 6 Department of Health Systems Science, Kaiser Permanente Bernard J. Tyson School of Medicine, Pasadena, California

**Keywords:** adverse perinatal outcomes, chronic maternal hypertension, HDFN, hemolytic disease, neonatal jaundice, obstetrical care, preterm birth

## Abstract

**Objective:**

Hemolytic disease of the fetus and newborn (HDFN) is associated with significant infant morbidity and mortality. Characteristics of pregnancies impacted by HDFN are not well understood. Therefore, this study examines maternal and infant characteristics based on HDFN status in a large, integrated health care system in the United States.

**Study Design:**

This was a population-based, retrospective cohort study of 464,711 pregnancies that received care at Kaiser Permanente Southern California (KPSC) hospitals from January 2008 to June 2022. HDFN cases were ascertained using a validated algorithm of structured and unstructured data elements. HDFN due to ABO alloimmunization alone was excluded. Adjusted odds ratios (aORs) derived from logistic regression were used to describe the association between maternal and infant characteristics and HDFN diagnosis as well as adverse perinatal outcomes. For rare events, Firth's bias-reduced logistic regression was applied.

**Results:**

A total of 136 HDFN pregnancies with 138 HDFN births (live births = 137; stillbirth = 1) were observed in the study. Of three twin pregnancies, all but one fetus had an HDFN diagnosis. HDFN diagnosis was associated with a maternal age of ≥35 years (aOR: 1.74; 95% confidence interval [CI]: 1.13–2.67), hypertension (2.07; 0.96–4.50), renal disease (3.43; 1.75–6.70), and multiparity (4.95; 2.73–8.95). Furthermore, HDFN diagnosis was associated with birth at 33 to 34 weeks (aOR: 5.72; 95% CI: 2.78–11.78) and 35 to 36 weeks (3.76; 2.38–5.94), and neonatal jaundice (3.11; 2.20–4.41). Birth weight ≥4,000 g was associated with lower HDFN diagnosis odds than normal weight (2,500–3,999 g; aOR: 0.36; 95% CI: 0.14–0.90). Hispanic race/ethnicity was associated with a lower HDFN diagnosis risk than non-Hispanic White (aOR: 0.63; 95% CI: 0.43–0.93).

**Conclusion:**

This study identified clinical and demographic factors linked with HDFN diagnosis, including specific maternal characteristics, medical/obstetrical factors, and neonatal factors, within a large, integrated health care system that can help inform management plans.

**Key Points:**

- Characteristics of HDFN are not well understood.
- This study examined HDFN characteristics in the United States.
- HDFN risk is linked to medical/obstetric factors.
- Increased risk of prematurity associated with HDFN.

Hemolytic disease of the fetus and newborn (HDFN) is a rare but serious condition caused by maternal alloimmunization against fetal red blood cells during pregnancy. 1 In the United States, the prevalence of HDFN ranges from 3/100,000 to 80/100,000 pregnancies annually. 2 Although HDFN incidence is declining with the availability of RhD immunoglobulins and the implementation of antibody screening programs, 3 4 HDFN continues to affect pregnancies in many developing nations and under-resourced settings that do not have universal screening and/or infrastructures, such as laboratory testing or advanced fetal monitoring systems, which are required to identify and manage HDFN. 5 6 7

Identification of HDFN during pregnancy is crucial so pregnant patients can be provided with proper clinical management and treatment. 8 Without proper screening and treatment, maternal antibodies can attack fetal red blood cells, leading to hemolysis and anemia. Therefore, severe HDFN cases can present antenatally as severe anemia, resulting in hydrops fetalis, and postnatally as hyperbilirubinemia. 1 Potential risk factors associated with HDFN include advanced maternal age at delivery, 9 non-Hispanic White race/ethnicity, 10 and a history of HDFN in a previous pregnancy. 11

Interventions for severely anemic fetuses include intrauterine blood transfusions (IUTs), which have significantly improved perinatal outcomes for decades. 12 However, IUTs are invasive procedures and have been associated with fetal morbidity, including posttransfusion cord bleeding, fetal bradycardia, premature rupture of membranes, emergency cesarean section, and fetal vascular accidents. 13 Since IUTs before the gestational age of 20 to 22 weeks are technically challenging to perform and increase the likelihood of these complications, clinicians may opt to administer intravenous immunoglobulin (IVIg) to delay or replace early IUT procedures. 14

Due to its rarity, HDFN has not been the subject of many studies; therefore, there is limited knowledge about its epidemiology and postnatal complications in the United States. Our objective was to evaluate the maternal and child demographic, medical, and obstetrical characteristics of pregnancies with HDFN compared with those without HDFN in a large, integrated health care delivery system in Southern California.

## Materials and Methods

### Study Setting

Data were extracted from the Kaiser Permanente Southern California (KPSC) electronic health records (EHRs). KPSC, a large, integrated health care system providing service to >4.8 million members across Southern California, 15 16 17 is broadly representative of the demographic and socioeconomic diversity of those living in Southern California. 15 KPSC EHRs contain detailed data for members, covering visits across all health care settings. Clinical care of KPSC members provided by external contracted providers (<3%) is captured in EHRs through insurance claim requests. The ethics committee of the Institutional Review Board of KPSC approved the study with an exemption for patient informed consent.

### Study Population

**Fig. 1:**
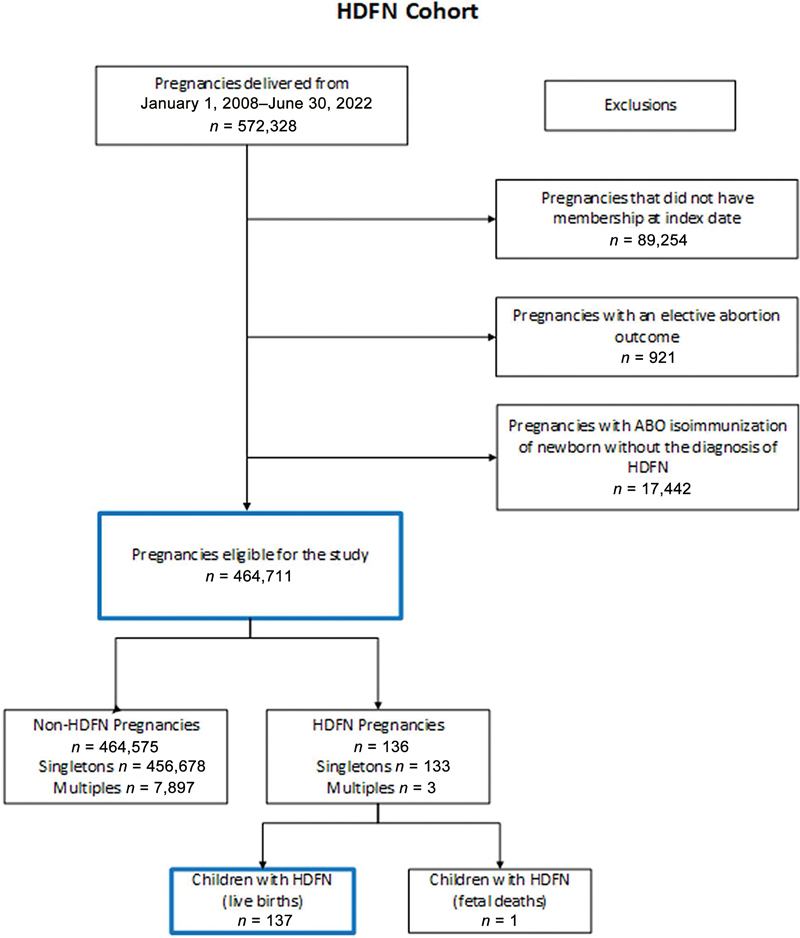
Hemolytic disease of the fetus and newborn (HDFN) cohort composition flowchart. Included ^a^ three twin pregnancies: five babies with HDFN babies and one baby without HDFN.

This was a population-based, retrospective cohort study of pregnant patients who received obstetrical care at the KPSC health care system from January 1, 2008, to June 30, 2022. We excluded pregnancies that (1) did not have membership at the start of the pregnancy (index date), (2) had an elective abortion outcome, and (3) had an ABO alloimmunization of the newborn alone without an HDFN diagnosis. After exclusions, we had 464,711 pregnancies eligible for the study. The study cohort composition is illustrated as a flowchart in Fig. 1 .

### Outcome: Identification of HDFN

The primary outcome, HDFN, was identified from KPSC EHRs based on an algorithm that was previously validated. 18 Briefly, the algorithm used “International Classification of Diseases, Ninth/Tenth Revisions, Clinical Modification” (ICD-9/10-CM) codes/clinical notes to identify potential pregnancies with a diagnosis of HDFN. 18 Then, the trained chart abstractors reviewed these records, examining structured (procedural/diagnostic codes) and unstructured data (clinical notes in the EHR for the mother or the infant), to confirm whether each record was a case with HDFN. Cases with HDFN had (1) mothers with antibodies demonstrating alloimmunization, (2) infants with positive direct antibody test (DAT) results, and (3) infants who received treatments such as phototherapy for jaundice, blood transfusion, or IVIg for anemia or infants with abnormal hemoglobin, hematocrit levels, or reticulocytes count results. HDFN was also marked where infants had negative DAT results but the mother received several IUTs. Unclear cases were adjudicated by our maternal-fetal medicine specialist (MJF). We also examined the frequency of IUTs among HDFN cases, calculating the proportion of pregnancies requiring IUTs. Additionally, we analyzed the timing and number of IUTs stratified by etiologic antibody to assess patterns in treatment. The secondary outcomes investigated in the study included fetal death, APGAR score <7 at 5 minutes, birth asphyxia, hypoxic-ischemic encephalopathy, neonatal jaundice, kernicterus, and cerebral palsy.

### Characteristics

Maternal characteristics evaluated in this study were age at index year, race/ethnicity (non-Hispanic White [White], non-Hispanic Black [Black], Hispanic, Asian/Pacific Islander, other/multiple, or unknown), household income (<$30,000, $30,000–49,999, $50,000–69,999, $70,000–89,999, ≥$90,000, or missing), and insurance type (Medicaid, commercial, private, or other). We also examined smoking, alcohol, or illicit drug use during pregnancy (yes/no), parity, gravidity, and prepregnancy body mass index (BMI; kg/m ^2^ ). For clinical characteristics, we examined gestational weight gain (lbs), medical (asthma, chronic hypertension, pregestational diabetes, renal disease, and autoimmune disease), and obstetrical (preterm premature rupture of membranes) comorbidities. For the child characteristics, we reported frequencies for the baby's sex, birth weight (g), gestational age at birth (weeks), head circumference (cm), preterm birth, fetal death, APGAR score of <7 at 5 minutes, birth asphyxia, hypoxic-ischemic encephalopathy, neonatal jaundice, kernicterus, and cerebral palsy.

### Statistical Analysis

We examined the distribution of maternal and child characteristics by HDFN status. For categorical variables, frequencies and percentages were estimated for each level and the distribution of each variable was compared using a chi-square test. For continuous variables, the means and standard deviations (SDs) were estimated and compared using *t-* tests. *p* -Values were 2-sided, and statistical significance was set at *p* <0.05. Logistic regression analysis was conducted to estimate the crude and adjusted odds ratios (aORs) for associations between maternal/fetal/infant characteristics and HDFN risk, reported as point estimates with 95% confidence intervals (CIs). We applied Firth's bias-reduced logistic regression for rare events. Statistical analysis was performed using SAS version 9.4 (SAS Institute, Cary, NC).

## Results

Of 464,711 KPSC pregnancies eligible for this study, a total of 136 pregnancies (29.3 cases per 100,000) with 138 births ( *n* = 137 live births; *n* = 1 stillbirth) were identified with HDFN ( Fig. 1 ). In the HDFN and non-HDFN groups, the mean (SD) ages were 31.8 (5.3) and 29.8 (5.7) years, respectively ( Table 1 ). Compared with non-HDFN pregnancies, HDFN pregnancies were more likely among older mothers (aged ≥30 years), patients of non-Hispanic White race/ethnicity, Medicaid-only insured patients, multipara patients, and multigravida patients. The HDFN group was composed of 38.2% ( *n* = 52) non-Hispanic White, 41.9% ( *n* = 57) Hispanic, 12.5% ( *n* = 17) Asian/Pacific Islander, and 7.4% ( *n* = 10) non-Hispanic Black patients. Among the HDFN group, there was a higher proportion of patients with chronic hypertension, renal disease, and a prepregnancy BMI of 30.0 to 34.9 kg/m ^2^ compared with the non-HDFN group.

**Table 1:** Distribution of maternal demographic, medical, and obstetrical characteristics based on hemolytic disease of the fetus and newborn (HDFN) status

Characteristic	Total ( *n* = 464,711)	HDFN status		
		HDFN ( *n* = 136)	Non-HDFN ( *n* = 464,575)	*p-* Value
Age at index date, y				
Mean (SD)	29.8 (5.7)	31.8 (5.3)	29.8 (5.7)	<0.0001 a
Age at index date, y, *n* (%)				
< 20	21,437 (4.6)	3 (2.2)	21,434 (4.6)	0.0030 b
20–29	193,523 (41.6)	40 (29.4)	193,483 (41.6)	
30–34	152,571 (32.8)	51 (37.5)	152,520 (32.8)	
≥35	97,180 (20.9)	42 (30.9)	97,138 (20.9)	
Race/ethnicity, *n* (%)				
Non-Hispanic White	126,026 (27.1)	52 (38.2)	125,974 (27.1)	0.0121 b
Non-Hispanic Black	36,344 (7.8)	10 (7.4)	36,334 (7.8)	
Hispanic	213,525 (45.9)	57 (41.9)	213,468 (45.9)	
Asian/Pacific Islander	62,045 (13.4)	17 (12.5)	62,028 (13.4)	
Other/multiple	5,794 (1.2)	0 (0.0)	5,794 (1.2)	
Unknown	20,977 (4.5)	0 (0.0)	20,977 (4.5)	
Household income, USD, *n* (%)				
< $30,000	17,307 (3.7)	4 (2.9)	17,303 (3.7)	0.8428 b
$30,000–$49,999	113,957 (24.5)	29 (21.3)	113,928 (24.5)	
$50,000–$69,999	135,554 (29.2)	42 (30.9)	135,512 (29.2)	
$70,000–$89,999	96,346 (20.7)	27 (19.9)	96,319 (20.7)	
≥$90,000	100,388 (21.6)	34 (25.0)	100,354 (21.6)	
Missing	1,159 (0.2)	0 (0.0)	1,159 (0.2)	
Insurance type, *n* (%)				
Medicaid	44,583 (9.6)	22 (16.2)	44,561 (9.6)	0.0351 b
Commercial	386,724 (83.2)	101 (74.3)	386,623 (83.2)	
Private	27,385 (5.9)	11 (8.1)	27,374 (5.9)	
Other/unknown	6,019 (1.3)	2 (1.5)	6,017 (1.3)	
Smoking during pregnancy, *n* (%)				
No	453,040 (97.5)	132 (97.1)	452,908 (97.5)	0.7487 b
Yes	11,671 (2.5)	4 (2.9)	11,667 (2.5)	
Alcohol use during pregnancy, *n* (%)				
No	403,999 (86.9)	120 (88.2)	403,879 (86.9)	0.6528 b
Yes	60,712 (13.1)	16 (11.8)	60,696 (13.1)	
Illicit drug use during pregnancy, *n* (%)				
No	446,864 (96.2)	131 (96.3)	446,733 (96.2)	0.9207 b
Yes	17,847 (3.8)	5 (3.7)	17,842 (3.8)	
Parity, *n* (%)				
Multiparous	264,887 (57.0)	120 (88.2)	264,767 (57.0)	<0.0001 b
Nulliparous	140,722 (30.3)	11 (8.1)	140,711 (30.3)	
Unknown	59,102 (12.7)	5 (3.7)	59,097 (12.7)	
Gravidity, *n* (%)				
Multigravida	326,956 (70.4)	126 (92.6)	326,830 (70.4)	<0.0001 b
Nulligravida	136,049 (29.3)	10 (7.4)	136,039 (29.3)	
Unknown	1,706 (0.4)	0 (0.0)	1,706 (0.4)	
Prepregnancy BMI, kg/m ^2^ , *n* (%)				
< 18.5	9,302 (2.0)	2 (1.5)	9,300 (2.0)	0.0505 b
18.5–24.9	172,300 (37.1)	49 (36.0)	172,251 (37.1)	
25.0–29.9	121,422 (26.1)	28 (20.6)	121,394 (26.1)	
30.0–34.9	68,732 (14.8)	25 (18.4)	68,707 (14.8)	
≥35.0	54,829 (11.8)	12 (8.8)	54,817 (11.8)	
Missing	38,126 (8.2)	20 (14.7)	38,106 (8.2)	
Gestational weight gain, lbs				
Mean (SD)	27.5 (15.88)	25.6 (15.73)	27.5 (15.88)	0.0376 a
Asthma, *n* (%)				
No	440,344 (94.8)	131 (96.3)	440,213 (94.8)	0.4122 b
Yes	24,367 (5.2)	5 (3.7)	24,362 (5.2)	
Chronic hypertension, *n* (%)				
No	455,405 (98.0)	130 (95.6)	455,275 (98.0)	0.0449 b
Yes	9,306 (2.0)	6 (4.4)	9,300 (2.0)	
Pregestational diabetes, *n* (%)				
No	458,543 (98.7)	136 (100.0)	458,407 (98.7)	0.1761 b
Yes	6,168 (1.3)	0 (0.0)	6,168 (1.3)	
Renal disease, *n* (%)				
No	456,372 (98.2)	128 (94.1)	456,244 (98.2)	0.0003 b
Yes	8,339 (1.8)	8 (5.9)	8,331 (1.8)	
Autoimmune disease, *n* (%)				
No	463,441 (99.7)	135 (99.3)	463,306 (99.7)	0.3020 b
Yes	1,270 (0.3)	1 (0.7)	1,269 (0.3)	
PPROM, *n* (%)				
No	458,085 (98.6)	133 (97.8)	457,952 (98.6)	0.4428 b
Yes	6,626 (1.4)	3 (2.2)	6,623 (1.4)	

Abbreviations: BMI, body mass index; PPROM, preterm premature rupture of membranes; SD, standard deviation; USD, United States dollar.

a Kruskal–Wallis *p* -values.

b Chi-square *p* -values.

Compared with infants born without a diagnosis of HDFN, infants born with a diagnosis of HDFN were more likely to be male, to be born preterm (delivery at <37 ^0/7^ weeks gestation), to have low birth weight (<2,500 g), to have a smaller head circumference, and to develop neonatal jaundice ( Table 2 ).

**Table 2:** Distribution of child characteristics and perinatal outcomes c based on hemolytic disease of fetus and newborn (HDFN) status

Characteristic	Total ( *n* = 446,499)	HDFN status		
		HDFN ( *n* = 138)	Non-HDFN ( *n* = 446,361)	*p* -Value
Sex, *n* (%)				
Female	216,646 (48.5)	59 (42.8)	216,587 (48.5)	0.3061 a
Male	228,476 (51.2)	79 (57.2)	228,397 (51.2)	
Missing	1,377 (0.3)	0 (0.0)	1,377 (0.3)	
Birth weight, g, *n* (%)				
< 1,500	5,982 (1.3)	4 (2.9)	5,978 (1.3)	0.0003 a
1,500–2,499	25,780 (5.8)	18 (13.0)	25,762 (5.8)	
2,500–3,999	366,607 (82.1)	110 (79.7)	366,497 (82.1)	
≥4,000	38,773 (8.7)	4 (2.9)	38,769 (8.7)	
Missing	9,357 (2.1)	2 (1.4)	9,355 (2.1)	
Gestational age at birth, wk, *n* (%)				
< 28	3,763 (0.8)	2 (1.4)	3,761 (0.8)	<0.0001 a
28–32	6,270 (1.4)	3 (2.2)	6,267 (1.4)	
33–34	8,765 (2.0)	12 (8.7)	8,753 (2.0)	
35–36	25,320 (5.7)	24 (17.4)	25,296 (5.7)	
≥37	402,381 (90.1)	97 (70.3)	402,284 (90.1)	
Head circumference, cm				
Mean (SD)	34.0 (2.7)	33.5 (2.3)	34.0 (2.7)	0.0332 b
Fetal death, *n* (%)				
No	444,315 (99.5)	137 (99.3)	444,178 (99.5)	0.6917 a
Yes	2,184 (0.5)	1 (0.7)	2,183 (0.5)	
APGAR score <7 at 5 min, *n* (%)				
No	435,606 (97.6)	135 (97.8)	435,471 (97.6)	0.3404 a
Yes	6,083 (1.4)	3 (2.2)	6,080 (1.4)	
Missing	4,810 (1.1)	0 (0.0)	4,810 (1.1)	
Birth asphyxia, *n* (%)				
No	446,441 (100.0)	138 (100.0)	446,303 (100.0)	0.8935 a
Yes	58 (0.0)	0 (0.0)	58 (0.0)	
Hypoxic-ischemic encephalopathy, *n* (%)				
No	445,887 (99.9)	138 (100.0)	445,749 (99.9)	0.6634 a
Yes	612 (0.1)	0 (0.0)	612 (0.1)	
Neonatal jaundice, *n* (%)				
No	283,942 (63.6)	48 (34.8)	283,894 (63.6)	<0.0001 a
Yes	162,557 (36.4)	90 (65.2)	162,467 (36.4)	
Kernicterus, *n* (%)				
No	446,494 (100.0)	138 (100.0)	446,356 (100.0)	0.9686 a
Yes	5 (0.0)	0 (0.0)	5 (0.0)	
Cerebral palsy, *n* (%)				
No	446,277 (100.0)	138 (100.0)	446,139 (100.0)	0.7933 a
Yes	222 (0.0)	0 (0.0)	222 (0.0)	

Abbreviation: SD, standard deviation.

a Chi-square *p* -value.

b Kruskal–Wallis *p* -value.

c Unit of analysis = live births and stillbirths.

Among the 136 pregnancies with HDFN, 17 (12.5%) required IUTs, while 117 (86.0%) did not require IUTs. The majority of IUTs were performed for cases with anti-D antibodies, with a mean gestational age of 27.6 weeks for the first transfusion ( Supplementary Table S1 , available in the online version only). The distribution of antibodies among HDFN cases with IUTs is shown in Supplementary Fig. S1 (available in the online version only).

Hispanic race/ethnicity was associated with a lower likelihood of HDFN diagnosis (aOR: 0.63; 95% CI: 0.43, 0.93) than non-Hispanic White race/ethnicity ( Table 3 ). Maternal characteristics associated with an increased risk for HDFN were maternal age of ≥35 years (aOR: 1.74; 95% CI: 1.13, 2.67), chronic hypertension (aOR: 2.07; 95% CI: 0.96, 4.50), renal disease (aOR: 3.43; 95% CI: 1.75, 6.70), and multipara (aOR: 4.95; 95% CI: 2.73, 8.95).

**Table 3:** Association between maternal characteristics and hemolytic disease of the fetus and newborn diagnosis (2008–2022 a )

Characteristic	OR (95%CI)	
	Crude	Adjusted b
Age at index date, c y		
< 20	0.77 (0.21, 2.77)	1.32 (0.38, 4.65)
20–29	1.00 (reference)	1.00 (reference)
30–34	1.47 (0.96, 2.27)	1.29 (0.84, 1.98)
≥35	2.15 (1.41, 3.28)	1.74 (1.13, 2.67)
Race/ethnicity		
Non-Hispanic White	1.00 (reference)	1.00 (reference)
Non-Hispanic Black	0.69 (0.36, 1.35)	0.66 (0.34, 1.28)
Hispanic	0.65 (0.44, 0.94)	0.63 (0.43, 0.93)
Asian/Pacific Islander	0.68 (0.39, 1.16)	0.65 (0.38, 1.10)
Other/multiple	0.21 (0.01, 3.36)	0.21 (0.01, 3.03)
Unknown	0.06 (0.00, 0.93)	0.04 (0.00, 0.65)
Household income, d USD		
< $30,000	0.76 (0.28, 2.02)	0.84 (0.32, 2.22)
$30,000–49,999	0.75 (0.46, 1.23)	0.85 (0.51, 1.41)
$50,000–69,999	0.91 (0.58, 1.43)	1.02 (0.65, 1.60)
$70,000–89,999	0.83 (0.50, 1.37)	0.89 (0.55, 1.46)
≥$90,000	1.00 (reference)	1.00 (reference)
Missing	1.26 (0.08, 20.47)	1.21 (0.08, 17.98)
Insurance type		
Medicaid	1.20 (0.59, 2.45)	1.30 (0.64, 2.67)
Commercial	0.62 (0.34, 1.15)	0.70 (0.39, 1.28)
Private	1.00 (reference)	1.00 (reference)
Other/unknown	0.99 (0.25, 3.88)	1.22 (0.32, 4.61)
Smoking status during pregnancy, yes	1.32 (0.52, 3.38) d	1.41 (0.57, 3.48)
Alcohol consumption during pregnancy, yes	0.91 (0.54, 1.52) d	1.05 (0.63, 1.73)
Illicit drug use during pregnancy, yes	1.05 (0.45, 2.46) d	1.38 (0.60, 3.13)
Prepregnancy BMI, kg/m ^2^		
< 18.5	0.94 (0.26, 3.33)	1.06 (0.31, 3.62)
18.5–24.9	1.00 (reference)	1.00 (reference)
25.0–29.9	0.82 (0.52, 1.30)	0.76 (0.48, 1.20)
30.0–34.9	1.29 (0.80, 2.08)	1.16 (0.72, 1.88)
≥35.0	0.79 (0.43, 1.48)	0.70 (0.37, 1.29)
Missing	1.87 (1.12, 3.13)	2.18 (1.31, 3.61)
Gestational weight gain, lbs	0.99 (0.98, 1.00)	0.99 (0.98, 1.01)
Comorbidities e		
Asthma	0.76 (0.32, 1.77)	0.75 (0.33, 1.70)
Chronic hypertension	2.44 (1.11, 5.36)	2.07 (0.96, 4.50)
Pregestational diabetes	Not estimated	Not estimated
Renal disease	3.62 (1.81, 7.25)	3.43 (1.75, 6.70)
Autoimmune disease	4.04 (0.81, 20.23)	3.88 (0.82, 18.27)
Parity		
Nulliparous	1.00 (reference)	1.00 (reference)
Multiparous	5.57 (3.04, 10.20)	4.95 (2.73, 8.95)
Unknown	1.14 (0.41, 3.15)	1.14 (0.43, 3.03)
Gravidity		
Nulligravida	1.00 (reference)	1.00 (reference)
Multigravida	5.01 (2.67, 9.41)	1.35 (0.53, 3.39)
Unknown	3.80 (0.22, 64.85)	2.88 (0.18, 45.30)
PPROM	1.81 (0.63, 5.24)	2.09 (0.75, 5.80)

Abbreviations: BMI, body mass index; CI, confidence interval; OR, odds ratio; PPROM, preterm premature rupture of membranes; USD, U.S. dollar.

a 2022 data were limited to 6 months.

b Adjusted for maternal race/ethnicity, age, household income, insurance type, prepregnancy BMI, and parity.

c Index date refers to the earliest date of pregnancy start.

d Median household income was based on the 2010 census tract of residence information.

e Comorbidities diagnosed within 1 year prior to the index date.

Similarly, a fetal and infant characteristic associated with an increased risk for HDFN was neonatal jaundice (aOR: 3.11; 95% CI: 2.20, 4.41). Compared with non-HDFN pregnancies, HDFN pregnancies were more likely to deliver preterm at gestation 33 to 34 weeks (aOR: 5.72; 95% CI: 2.78, 11.78) and 35 to 36 weeks (aOR: 3.76; 95% CI: 2.38, 5.94) than at term gestation. Meanwhile, lower odds of HDFN were associated with a birth weight ≥4,000 g compared with a normal weight (2,500–3,999 g; aOR: 0.36; 95% CI: 0.14, 0.90; Table 4 ).

**Table 4:** Association between fetal and infant characteristics and hemolytic disease of the fetus and newborn diagnosis a (2008–2022 b )

Characteristic	OR (95% CI)	
	Crude	Adjusted c
Sex		
Male	1.00 (Reference)	1.00 (Reference)
Female	0.79 (0.56, 1.10)	0.79 (0.57, 1.09)
Missing/unknown	Not estimated	Not estimated
Birth weight, g		
< 1,500	2.50 (0.97, 6.41)	1.64 (0.39, 6.82)
1,500–2,499	2.38 (1.46, 3.90)	1.00 (0.53, 1.85)
2,500–3,999	1.00 (Reference)	1.00 (Reference)
≥4,000	0.38 (0.15, 0.99)	0.36 (0.14, 0.90)
Missing/unknown	0.89 (0.25, 3.11)	1.10 (0.33, 3.71)
Gestational age at birth, wks		
< 28	2.74 (0.78, 9.63)	2.33 (0.42, 13.07)
28–32	2.30 (0.79, 6.69)	2.06 (0.55, 7.71)
33–34	5.89 (3.27, 10.62)	5.72 (2.78, 11.78)
35–36	4.00 (2.57, 6.22)	3.76 (2.38, 5.94)
≥37	1.00 (Reference)	1.00 (Reference)
Head circumference, cm	0.91 (0.85, 0.97)	0.97 (0.88, 1.07)
Fetal death	2.23 (0.45, 11.11)	1.55 (0.30, 8.03)
APGAR score <7 at 5 min	1.85 (0.64, 5.35)	1.25 (0.41, 3.86)
Birth asphyxia	Not estimated	Not estimated
Hypoxic-ischemic encephalopathy	Not estimated	Not estimated
Neonatal jaundice	3.26 (2.30, 4.62)	3.11 (2.20, 4.41)
Kernicterus	Not estimated	Not estimated
Cerebral palsy	Not estimated	Not estimated

Abbreviations: CI, confidence interval; OR, odds ratio.

a Estimates by HDFN status are presented using data on all births (live births and stillbirths).

b 2022 data were limited to 6 months.

c Adjusted for maternal race/ethnicity, age, household income, insurance type, prepregnancy BMI, parity, infant sex, birth weight, and gestational age at birth.

## Discussion

This population-based, retrospective cohort study reported the characteristics of HDFN-affected pregnancies compared with non-HDFN pregnancies using 14 years of data (January 1, 2008, to June 30, 2022) from KPSC hospitals. HDFN-affected pregnancies were relatively rare, with a prevalence rate of 29.3 per 100,000, consistent with previously reported rates. 2 19 While one study using the National Hospital Discharge Survey reported a higher prevalence 20 ; however, that study examined HDFN among live births spanning from 1996 to 2010 and may not reflect the declining rates of HDFN over the last few decades.

Consistent with previous research, 11 20 non-Hispanic White patients had a higher proportion of HDFN pregnancies (38% of HDFN cases) in our study. This is an important finding as the KPSC health care system has a racially and ethnically diverse patient population. 15 Furthermore, our study demonstrated that advanced maternal age and multipara status were associated with an increased risk of HDFN diagnosis. A previous case-control study 9 in a Dutch population found the opposite, that younger patients were more likely to have RhD immunization risk, but the authors noted that this was difficult to explain and that it could have been an artifact of their study design. The higher rates of HDFN among those with a higher prepregnancy BMI, chronic hypertension, and renal disease in our study should be investigated further. Finally, HDFN pregnancies in our analysis were associated with preterm birth and neonatal jaundice. This finding is also consistent with prior studies. 21 22 Preterm delivery could also be a result of antenatal therapy due to HDFN identified during pregnancy; it has been proposed that a medically indicated late preterm or early-term birth could reduce the fetal risk of ongoing exposure to maternal autoantibodies. 21 22

The study highlights the importance of monitoring pregnancies at risk for HDFN, particularly among patients who are older, multipara, and living with chronic health conditions. The association of HDFN with preterm birth and neonatal jaundice underscores the need for targeted prenatal care and possible early intervention strategies. Our findings indicate that while the majority (86.0%) of pregnancies affected by HDFN did not require transfusion, those with certain alloantibodies, particularly anti-D, were more frequently given IUTs. The mean timing of the first transfusion varied by antibody type. Further research is needed to confirm these findings and establish comprehensive guidelines for managing HDFN in diverse clinical settings. Additional studies should evaluate the risk of subsequent HDFN in pregnancies previously affected by the condition and explore underlying mechanisms. Future research should focus on the long-term outcomes of infants born with HDFN and the effectiveness of different antenatal therapies in reducing HDFN-related complications.

## Strengths and Limitations

A major strength of this study was the ability to identify and examine 136 pregnancies with HDFN, a rare disorder in the United States. HDFN was evaluated among a large sample of a racially and ethnically diverse population, and HDFN cases were ascertained with a validated algorithm that combined structured and unstructured EHR data and confirmed them to be HDFN cases using chart review. 18 The use of KPSC EHRs allowed for the examination of comorbidities, and the linked datasets between the mother and fetus/neonate allowed for a rich examination of the distribution of HDFN compared with non-HDFN pregnancies among the mother and their baby spanning over a decade.

This study had some limitations. First, due to the rarity of this condition, the sample size after stratification was small for many characteristics. Thus, our point estimates may be underpowered, reducing the chance of detecting a true effect. Second, those who had a history of HDFN prior to receiving care at KPSC hospitals were not observed in this study.

The findings of this study demonstrated that HDFN was more common among non-Hispanic White patients, older mothers, multiparous patients, and those with chronic hypertension or renal disease. Newborns with HDFN had higher rates of preterm delivery and neonatal jaundice. These findings suggest that pregnancies affected by HDFN merit continued clinical attention.
