# Supplementary material for: Hemolytic Disease of the Fetus and Newborn in an Integrated Health Care System
Source: Am J Perinatol. 2025 Apr 17;42(15):2038–48. doi: 10.1055/a-2558-7891 (PMC12530894; doi:10.1055/a-2558-7891)
Supplement: Supplementary file 1 — Supplementary Material [file 10-1055-a-2558-7891-s24dec0779.pdf]

Supplementary Table S1 Distribution of antibody types among hemolytic disease of fetus and newborn (HDFN) pregnancies with intrauterine transfusions (IUTs)

Antibody type	*n* (HDFN pregnancies with IUTs)	Number of IUTs	Mean timing of 1st IUT in gestational weeks
Anti D	11	32	27.6
Anti Kell	3	7	30.0
Anti little c	1	1	32.0
Anti D, Anti C	1	2	21.0
Anti D, Anti E	1	2	31.0
Total	17	44	–

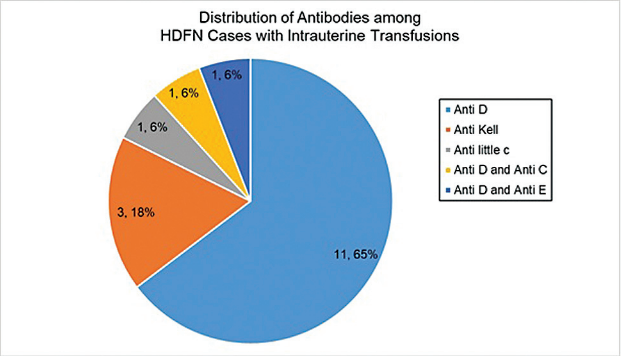

Supplementary Fig. S1 Distribution of antibodies among hemolytic disease of the fetus and newborn (HDFN) cases with intrauterine transfusions.

 © 2025. The Author(s).
